# Identifying genes underlying parallel evolution of stromal resistance to placental and cancer invasion

**DOI:** 10.1038/s41540-025-00577-z

**Published:** 2025-08-22

**Authors:** Yasir Suhail, Wenqiang Du, Junaid Afzal, Günter P. Wagner, Kshitiz

**Affiliations:** 1 Department of Biomedical Engineering, UConn Health, Farmington, CT USA; 2 Systems Biology Institute, Yale University, New Haven, CT USA; 3 Department of Medicine, University of California San Francisco, San Francisco, CA USA; 4 Department of Ecology and Evolutionary Biology, Yale University, New Haven, CT USA

**Keywords:** Cancer, Evolution, Systems biology

## Abstract

Stromal regulation of cancer dissemination is well recognized, however causal genes remain unidentified. We previously demonstrated that epitheliochorial species have acquired stromal resistance to placental invasion, correlating with reduced rate of cancer malignancies, identifying stromal genes correlating with depth of placental invasion called ELI (Evolved Levels of Invasibility) genes. Similarly, decidualization of human endometrial fibroblasts confers resistance to placental invasion. We hypothesized that both trajectories may convergently use similar pathways, providing an opportunity to identify stromal genes regulating epithelial invasion. We created a gene-set ELI-D1 (ELI-Decidual 1), putatively underlying stromal vulnerability to invasion. ELI-D1 were negatively enriched in T1-T2 stage transition in many human cancers, typically preceding dissemination. We also identified candidate transcriptional regulators underlying variation in ELI-D1 genes across eutherians, functionally showing Nr2f6, and JDP2 can regulate stromal resistance to invasion in human fibroblasts. Our comparative approach provides us with a gene-set linked to stromal vulnerability in human cancers.

## Introduction

**Fig. 1:**
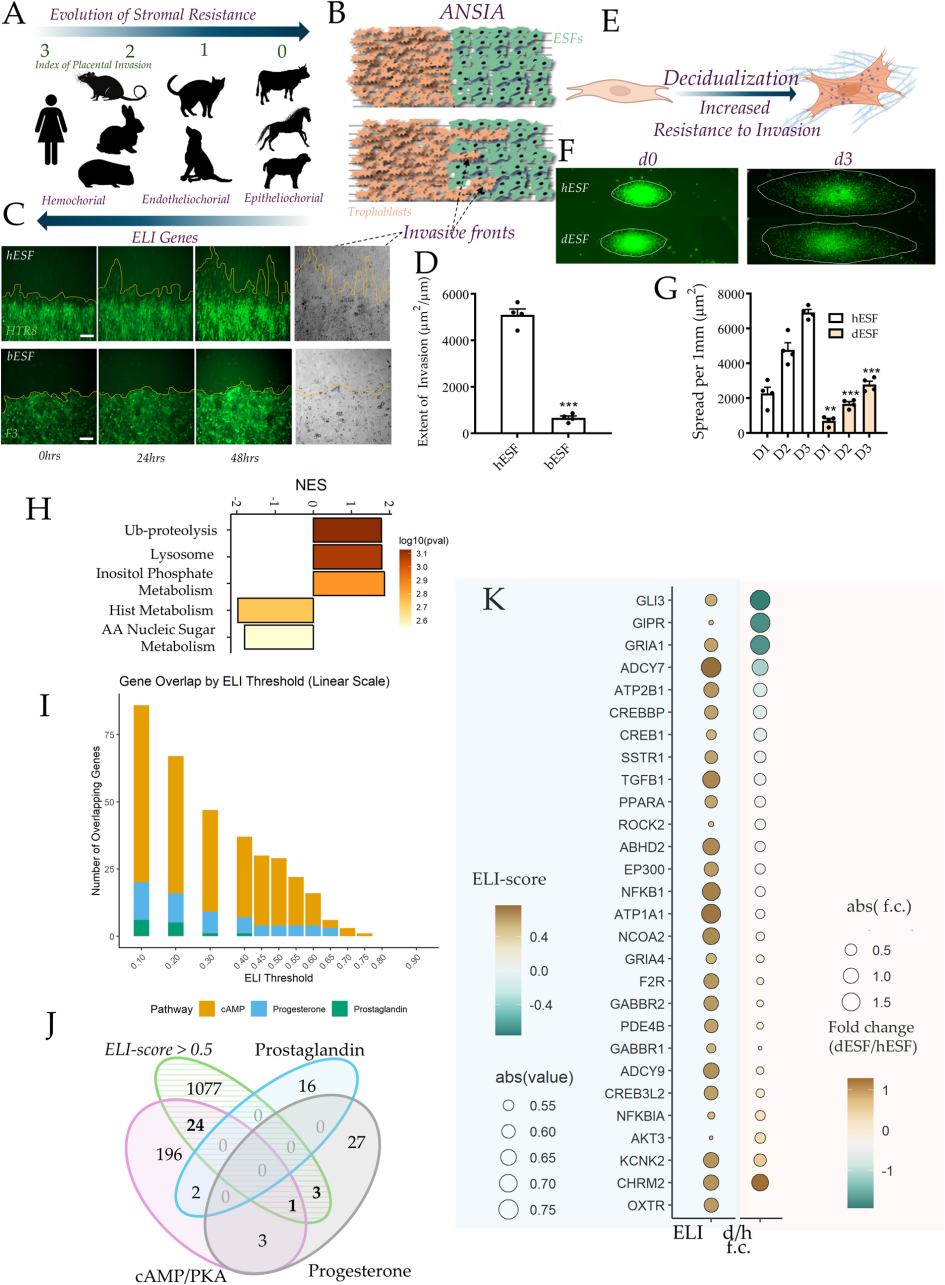
Identification of evolved stromal genes contributing to vulnerability to placental and cancer invasion. **A**–**G** Epitheliochorial and decidual ESFs in hemochorial species are characterized by high resistance to trophoblast invasion. **A** aESFs from species in our analysis to identify ELI genes in stroma correlating with placental invasion; Shown is the placental invasion index (0,1,2,3) with increasing number referring to higher placental invasion into the endometrial stroma; Stromal resistance has evolved to increase in epitheliochorial species (top arrow); ELI genes (bottom arrow) are calculated to be higher in stroma of species with deeper invasion. **B** ANSIA quantitatively measures stromal invasion of trophoblasts, with **(C)** stills from invasion of HTR8 into hESFs, and F3 into bESFs, **(D)** Quantification of the extent of invasion; *n* = 4 samples. **E** ESF decidualization results in increased stromal resistance; **F** HTR8 spheroid invasion on anisotropic hESF, and decidualized hESFs (dESFs) monolayers, quantified at 24 h (D1), 48 h (D2), and 72 h (D3) (**G**); *n* = 4 samples**. H–K Evolved stromal vulnerability gene-set:ELI-D1:** **H** Pathway enrichment in ELI genes; **I** Venn diagram showing overlap of key decidual pathways and gene counts with ELI score> 0.5; **J** Histogram showing number of identified genes in the ELI-D1 gene-set based on thresholding of ELI score; **K** ELI-score (left), and fold-change in decidualization for ELI-D1 genes.

Placentation substantially varies across mammals, from deeply invasive (hemochorial) to non-invasive (epitheliochorial). This variation is also correlated with differences in rates of cancer malignancy rates in these mammals^1^. We have explained the connection between placental and cancer invasion in an evolutionary framework called **Evolved Levels of Invasibility (ELI)**, showing that endometrial fibroblasts (ESFs) have evolved in epitheliochorial species to resist placental invasion^2^ (Fig. 1A). ELI states that reduced cancer metastasis in epitheliochorial species is a secondary, systematic effect of this phenomenon, likely due to correlated evolution of stroma across other tissues^3,4^. We have also shown that ELI genes, which are derived from the comparative stromal evolution across epitheliochorial and hemochorial mammalian stroma, are significantly prognostic in predicting survival in human melanoma patients^5^. Identifying the stromal genes that have evolved to confer resistance, or vulnerability to invasion from epithelia will provide new opportunities to mechanistically understand desmoplastic reaction, as well as develop stroma-targeted therapies to manage cancer metastasis. This objective is however challenging to achieve, because differential gene expression between distant species reveals thousands of genes, most unrelated to any given phenotype. Further, pooled genome-wide gene perturbation libraries are not feasible for an enrichment of a collective phenotype of invasibility.

We therefore identified stromal genes that have changed along with evolution of stromal resistance at the maternal fetal interface with another parallel process at the same site, decidualization of endometrial stroma. Within hemochorial species, ESFs differentiate into a decidual state, which is evolutionarily adapted from the fibroblast activation response to the wounding of endometrial epithelium by implantation^6^, a process similar to the wounding of basal lamina by a growing tumor. Crucially, we have found that decidualization is a differentiation process, which markedly reduces trophoblast invasion by increased matrix production^7^, and actomyosin force generation^8^. Locally, decidualization can therefore be understood as an endometrium specific adaptation to regulate trophoblast invasion.

We therefore identified stromal genes that have changed along with the evolution of epitheliochorial placentation, while belonging to the key decidualization pathways—based on the assumption that both trajectories may have converged onto similar pathways to achieve stromal resistance. Our approach has identified limited number of genes putatively regulating invasibility which could be confirmed without need for a screen for discovery. This gene-set termed ELI-D1 (ELI and Decidual combined 1) primarily contains negative regulators of cAMP/Protein Kinase A pathway, and progesterone signaling. Interestingly, ELI-D1 was negatively enriched in many TCGA (The Cancer Genome Atlas) cancer genes associated with T1 to T2 stage transition which precedes stromal trespass, or the dissemination of cancer cells beyond the basal lamina into the stromal compartment. We identified the transcription factor binding sites that had changed in the promoter regions of ELI-D1 genes, correlating their copy numbers with the downstream gene expression in endometrial fibroblasts across 9 eutherian mammals. These transcription factors are likely to have contributed to the pattern expression of ELI-D1 genes across mammals. Finally, using a nanopatterned assay to quantitatively measure stromal regulation of invasion, we confirm the most likely transcription regulators of a plurality of ELI-D1 genes, Nr2f6, and JDP2 to be causal in conferring increased vulnerability to invasion in human dermal fibroblasts, as well as cancer associated fibroblasts.

## Results

### Identifying stromal gene candidates explaining vulnerability to placental invasion in humans

Previously, using RNAseq data from ESFs isolated from 9 eutherian mammals, we had identified ELI genes whose expression in ESFs correlate with increased placental invasion (Fig. 1A)^9^. ELI genes are expressed correlatively more in hemochorial species vs epitheliochorial species characterized by little to no invasion of the placental trophoblasts into the endometrial stroma. We used Accelerated Nanopatterned Stromal Invasion Assay (ANSIA), which we had previously developed to measure stromal resistance (or vulnerability) to invasion^2^ to invasion of Cytotracker green labeled HTR8 (human extravillous trophoblasts derived), and F3 (bovine trophoblast derived) into monolayers of human and bovine ESFs (Fig. 1B, C). bESFs completely resisted invasion of F3 over 48 hours, while hESFs were vulnerable to HTR8 invasion, which formed deep invasive forks into the stromal compartment (Fig. 1C, D). Among hemochorial species, the endometrium prepares for placentation by undergoing a profound transformation called decidualization (Fig. 1E), which results in increased resistance to trophoblast invasion^8,10–12^. We confirmed that decidualization also increases hESF resistance to HTR8 spheroid invasion on anisotropic monolayers of hESF, undifferentiated or decidualized (dESFs) prior to experimentation (Fig. 1F, G). Therefore, both the evolution of epitheliochorial placentation, as well as decidualization were associated with large, and significant increase in ESF resistance to corresponding trophoblast invasion.

ELI genes were previously identified by differential transcriptomic analysis of ESFs from nine different mammalian species in correlation with a placentation invasion index^3^. Gene-set enrichment revealed pathways related to proteasomal or lysosomal proteolysis, and metabolism, which although interesting, has an unclear connection to stromal resistance (Fig. 1H). We hypothesized that parallel accomplishment of similar stromal resistance in (i) evolution of epitheliochorial placentation, and (ii) decidualization of ESFs may converge on similar pathways. We therefore looked for significant ELI genes (ELI score > 0.5) that are part of the pathways critical for decidualization: Prostaglandin biosynthesis, cAMP/Protein Kinase A signaling, and Progesterone signaling (Fig. 1I, J). Among the three decidual gene sets, 28 genes overlap with ELI genes, 24 of them are regulators of PKA signaling, and 4 belonged to Progesterone biosynthesis. We call this gene-set ELI-D1 (ELI-Decidual Gene Set 1) and found that they also showed reduced expression in hESF post decidualization (Fig. 1K).

### Human cancers negatively enrich ELI-D1 genes in stage transition preceding metastasis

**Fig. 2:**
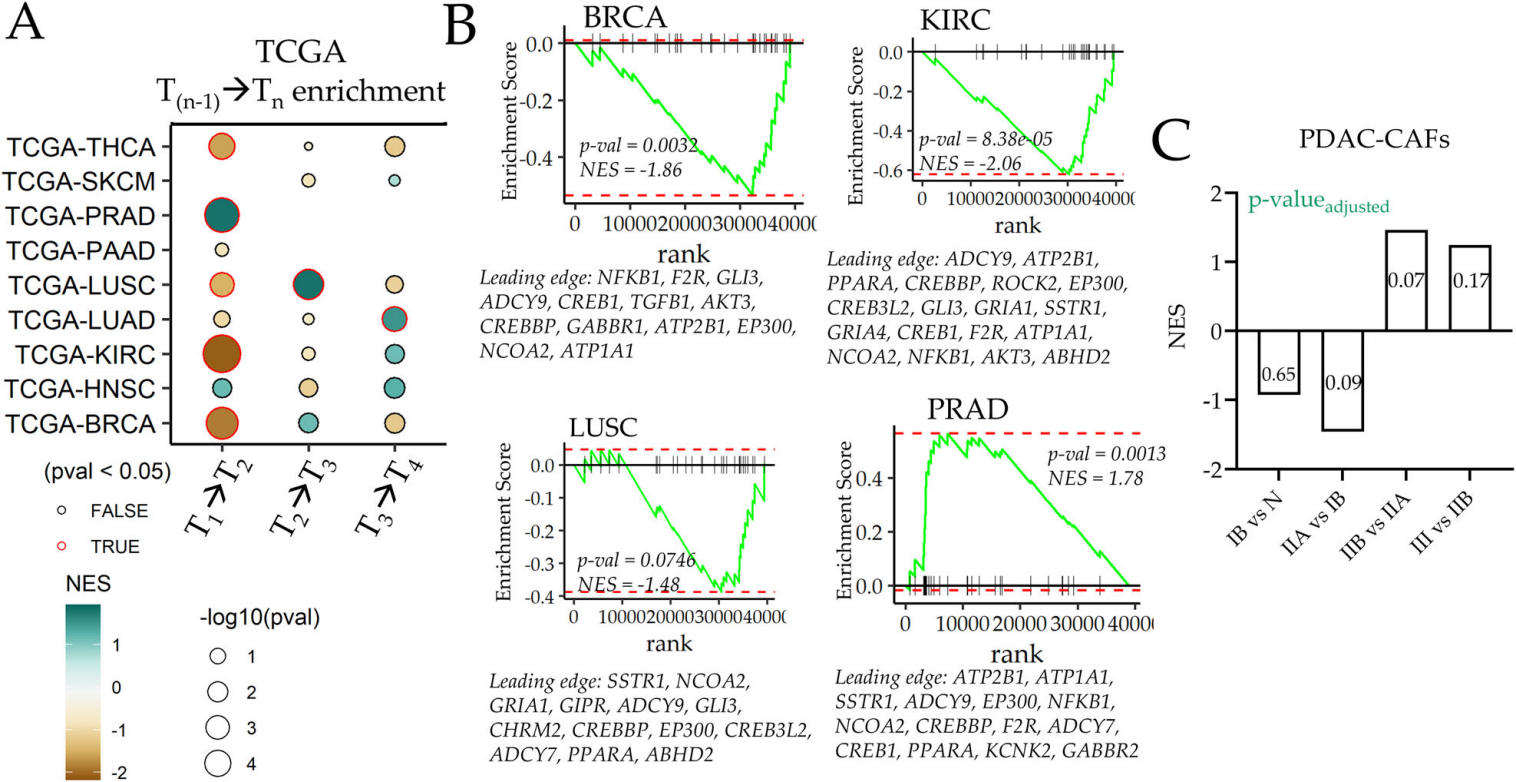
ELI-D1 enrichment in stage transitions in TCGA cancers. **A** Enrichment analysis of ELI-D1 in TCGA cancer-types stage transitions; size of bubbles reflect *p*-value, color shows enrichment score. **B** GSEA plots showing leading edge ELI-D1 genes in T1- > T2 transitions of selected TCGA cancers types. **C** ELI-D1 enrichment in fibroblasts from scRNAseq in PDAC patients at different stage transitions; *p*-value in bars.

We bioinformatically tested the hypothesis that ELI-D1 gene-set is linked to stromal vulnerability to invasion using stage-wise stratified data in The Cancer Genome Atlas (TCGA). GSEA for ELI-D1 genes across stages for many solid cancers showed significant negative enrichment, particularly in the T1 to T2 transition, which characterizes growth of tumor size, frequently preceding onset of dissemination. For most solid TCGA cancers, ELI-D1 was negatively enriched in the T1- > T2 transition suggesting that the putative pro-invasable effect of ELI-D1 precedes cancer dissemination. The leading edge genes for various cancers were ABHD2, ADCY7, ADCY9, AKT3, ATP1A1, ATP2B1, CHRM2, CREB1, CREB3L2, CREBBP, EP300, F2R, GABBR1, GIPR, GLI3, GRIA1, NCOA2, NFKB1, PPARA, SSTR1, TGFB1 (Fig. 2B). Pancreatic adenocarcinoma (PRAD) uniquely showed an opposite trend, wherein the T1- > T2 transition, as defined in TCGA annotation, was positively enriched. To test deeper, we used previously published scRNAseq data of 27 PRAD patients^13^, which was resolved for sub-staging and allowed testing the enrichment only in fibroblasts. We computed the ELI-D1 gene enrichment only within CAFs during stage transitions, finding that ELI-D1 genes were negatively enriched in stage II- > IIA, and then positively enriched for IIA- > IIB. It is the latter transition, IIA- > IIB in PRAD, which is associated with cancer dissemination to proximal lymph nodes, showing a consistent pattern across multiple cancer types for negative ELI-D1 enrichment accompanying the transition to a disseminating stage (Fig. 2B, C). Onset of dissemination is the first step in the metastatic cancer, therefore, the enrichment of ELI-D1 gene set across multiple cancer types prior to that stage indicates role of these genes in stromal transformation which accompanies onset of cancer dissemination.

### Candidate transcription factors regulating comparative expression of ELI-D1

**Fig. 3:**
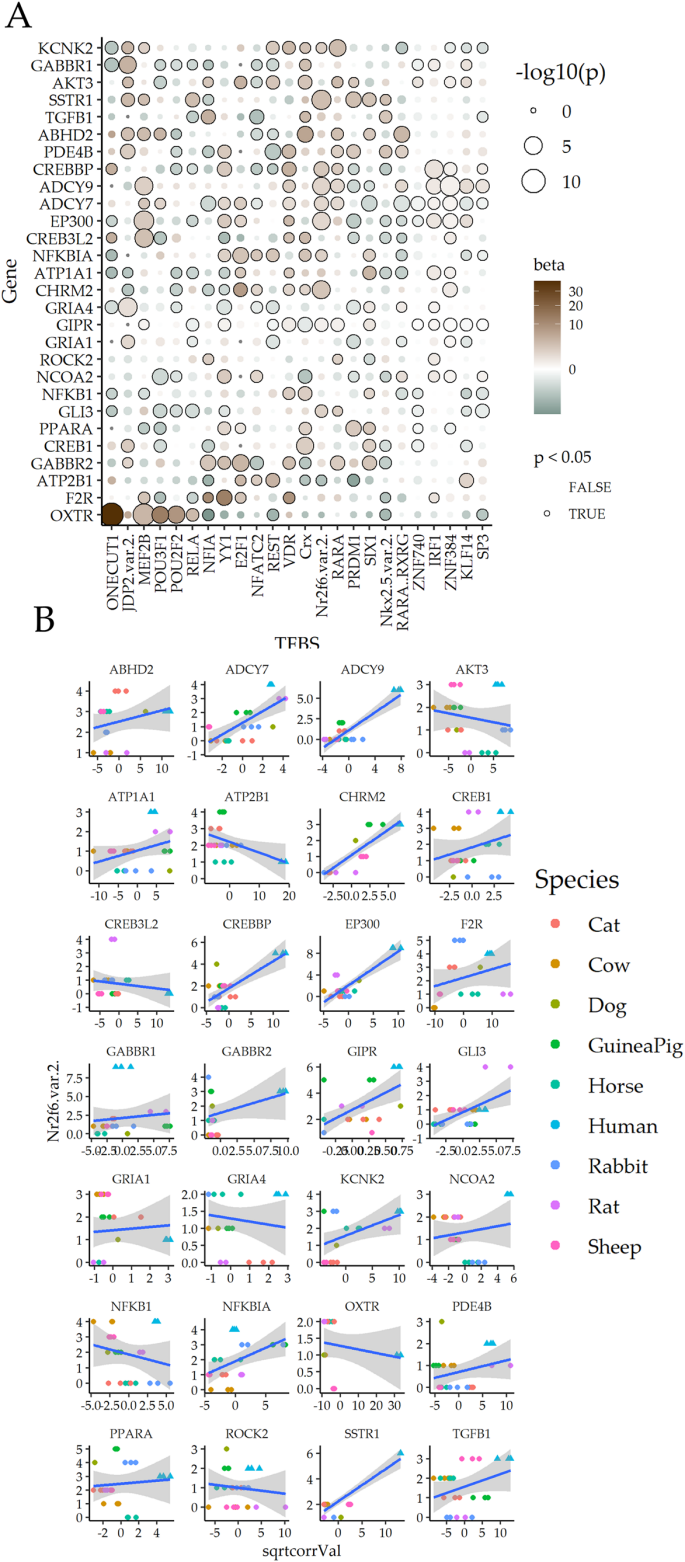
Transcription factor (TF) candidates regulating ELI-D1 gene expression across placental mammals. **A** TFs β-values explaining ELI-D1 variance across species based on copy number of TF binding sites in cis-regulatory regions of a given ELI-D1 gene (y-axis); TFs are shown in x-axis; significant correlation of TF binding site copy number and gene expression across 9 species shown in solid boundary to the bubble. **B** Example of a selected TF: Nr2f6 on variation of gene expression for each of the ELI-D1 genes across species; y-axis: copy number of Nr2f6; x-axis: square root of change in expression vs mean expression in 9 selected species.

Changes in the TF binding sites (TFBS) is a common mechanism of evolution of gene regulation^14^. TFBS changes, both in copy numbers as well as those affecting binding affinity of TFs to the mutated TFBS, can have a dramatic effect in the regulation of downstream gene expression. As a simple linear model, we calculated the regression coefficient β, a measure of the correlation of TFBS copy number in cis-regulatory region of each ELI-D1 gene with its expression^3^ (Fig. 3A). While there was no overarching single TF regulating comparative ELI-D1 expression, many significant ones informed expression of many leading-edge genes, particularly Nr2f6 and JDP2 showing ELI-D1 gene expression variation across the 9 eutherian species (Fig. 3B).

### Evolved transcriptional regulation of Nr2f6 and JDP2 confer stromal vulnerability to cancer invasion in human fibroblasts

**Fig. 4:**
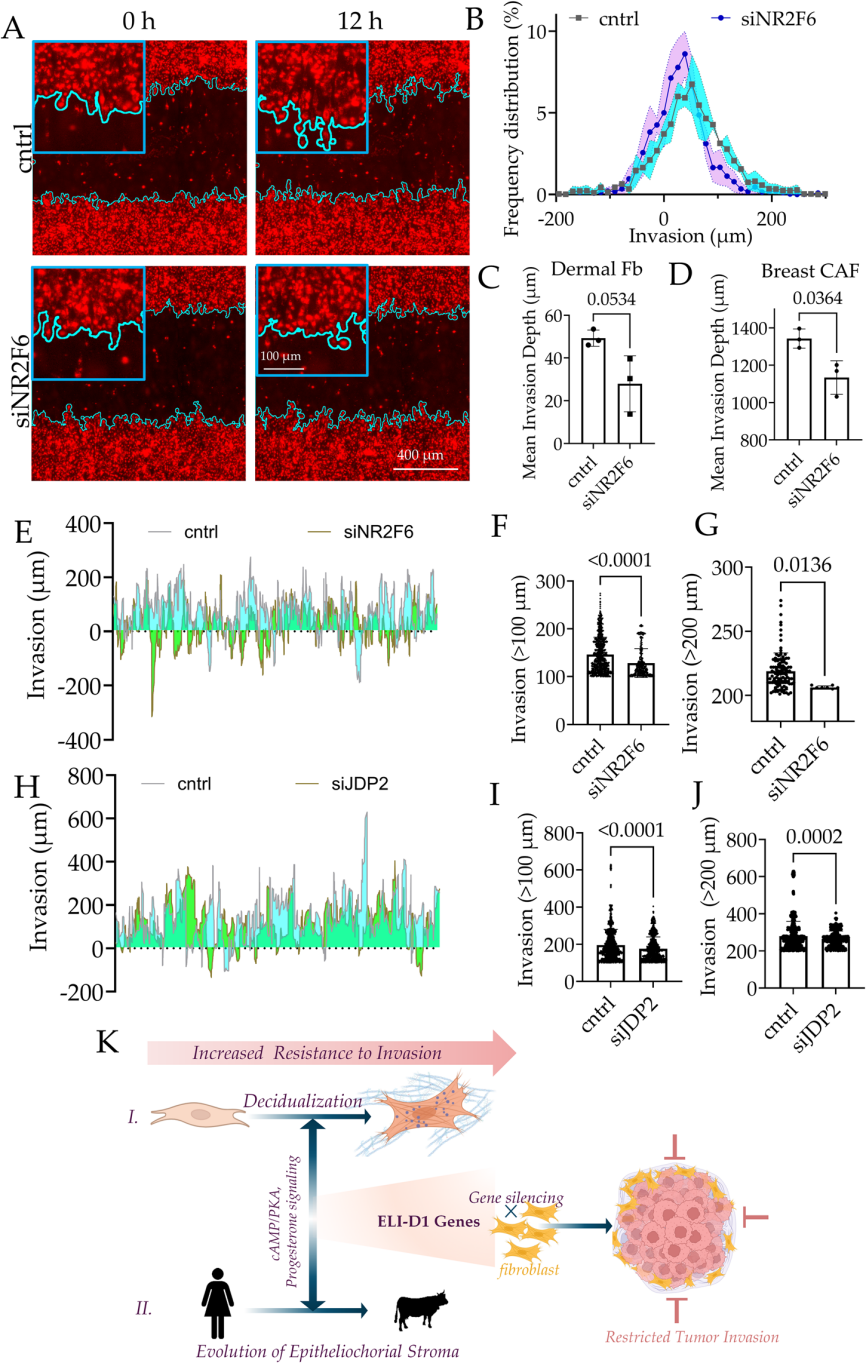
Silencing of predicted TF regulators of ELI-D1 genes confer stromal resistance to cancer invasion. **A** Fluorescent images of A375 (red) invading human dermal fibroblasts (black, unlabeled region). Overlayed lines show A375 invading fronts; Inset showing zoomed in invading fronts. **B** Histogram showing frequency distribution of A375 invasive forks of varying depth into the Dermal fibroblast compartment: control (scrambled) and NR2F6 silenced. **C, D** Graph showing mean A375 invasion depth from three independent experiments into scrambled and NR2F6 silenced Dermal fibroblasts (**C**), as well as CAFs isolated from breast cancer (**D**). **E** A375 invasion across different horizontal positions in a field of view for control, and NR2F6 silenced Dermal Fibroblasts; **F, G** Graph showing invasion depth deeper than 100 µm and 200 µm. For scrambled and siNR2F6, *N* = 1092 and 344 in **(F)**; *N* = 112 and 8 in **(G)**, respectively. **H** A375 invasion across different horizontal positions in a field of view for control, and JDP2 silenced Dermal Fibroblasts; **I, J** Graph showing invasion depth deeper than 100 µm and 200 µm. For scrambled and siJDP2, *N* = 1926 and 1642 in **(I)**; *N* = 723 and 450 in **(J)**, respectively. **K** Schematic showing identification of ELI-D1 gene sets from two distinct phenotypically similar stromal phenomena: I. decidualization of endometrial fibroblasts, and II. Evolution of epitheliochorial stromal fibroblasts. Both the processes confer increased stromal resistance to trophoblast invasion. ELI-D1 genes are negative regulators, silencing their expression in fibroblasts is predicted to phenocopy increased stromal resistance, which could endow fibroblasts to increase resistance to invasion from trophoblasts, or cancer.

Our analysis showed that more than one TF likely played a role in regulating the differential expression of stromal genes across mammals regulating invasion. We chose Nr2f6, and JDP2 which had a broad and significant effect on a large subset of ELI-D1 gene expression across species (Fig. 4A), and tested their role in regulating stromal invasion. Towards this, we used ANSIA to measure the effect of NR2F6 gene silencing on human dermal fibroblasts resistance to invasion of A375, a cell line derived from malignant melanoma. NR2F6 silencing resulted in significantly reduced invasion of A375 into the perturbed human dermal fibroblasts (Fig. 4B, C), as well as in cancer associated fibroblasts isolated from breast tumor (Fig. 4D). Frequency distribution of invasive forks into dermal fibroblasts monolayer showed a reduction in deep invasive forks (Fig. 4B, E). Comparison of deeply invasive forks (>100 um) and (>200 um) showed that NR2F6 silenced cells were significantly more resistant to deep invasion (Fig. 4F, G). In particular, we noted very few high depth invasion of the invading A375 forks (>200 um) in dermal fibroblasts silenced for NR2F6 gene (Fig. 4G).

A similar trend was observed when dermal fibroblasts were silenced for JDP2, a TF which informed expression of many ELI-D1 genes across the hemochorial to epitheliochorial species (Fig. 3A). Dermal fibroblasts silenced for JDP2 showed a significantly different profile of A375 invasive front (Fig. 4H), with significantly lower number and depth of invasion for highly invasive forks (Fig. 4J).

## Discussion

We had previously reported that epitheliochorial placentation is primarily due to the evolution of stromal resistance to invasion, correlating with decreased cancer malignancy^2^. Using ESFs from 9 species, we have also identified stromal genes correlating with their invasibility, termed ELI genes^3,5^. As differential expression is not sufficient to link genes to phenotypes in distant species, we hypothesized that decidualization induced resistance in ESFs may use similar pathways. The identified ELI-D1 gene-set mostly contains inhibitors of cAMP/PKA pathway, and Progesterone biosynthesis pathway, not obvious candidates based on gene prioritization. Indeed, both these pathways are not well described in the literature for stromal regulation of cancer metastasis. Being limited, they can be easily validated experimentally without the need of a screen.

As a means of translational validation, we found that ELI-D1 gene set is negatively enriched for many human cancers preceding onset of their transition to metastasis. Surprisingly, while this onset was consistently enriched for many cancer types, pancreatic cancer showed an opposite trend. We therefore chose a previously published scRNAseq dataset from 27 patients with pancreatic ductal cell carcinoma, allowing a direct test for the enrichment in fibroblasts (as against bulk tumor sample in TCGA), as well as a more resolved staging. Indeed, stage II is sub-divided in stage IIA and IIB, with the latter associated with dissemination. These were sparse data (with few cancers at those stages), therefore statistically low powered, but the enrichment trend in the metastatic IIA to IIB transition was similar to those computed in other TCGA cancers.

CAFs are known to mount a resistive response to growing tumor, which when fails to resist, permits cancer to trespass stroma. ELI-D1 genes are significantly enriched across many cancers in this transition. Finally, we also found several putative candidate transcriptional regulators of evolved stromal vulnerability. We also functionally showed that Nr2f6 and JDP2 may contribute to differential expression of many ELI-D1 genes, and may contribute to increased stromal vulnerability to placental, and cancer malignancy in hemochorial vs epitheliochorial species.

Stromal contribution to placental and cancer invasion is widely appreciated, but search for underlying causal genes is still on, primarily as genotype-phenotype correlation necessitates large data, yet unavailable. Our ELI hypothesis explaining evolution of stromal resistance at MFI, a site of high genetic selectivity and phenotypic variance across mammals has revealed genes that contribute to the inception of cancer dissemination, opening opportunities for anti-metastatic stroma-targeted therapeutics (Fig. 4K).

## Methods

### Bioinformatics

ELI scores were assigned to genes based on previously described method^3,5^. Pathway enrichment was computed using Gene Set Enrichment Analysis^15^. Cancer patient population gene expression data and staging was downloaded from TCGA^16^, and gene set enrichment analysis performed for each transition stages^15^. To count transcription factor binding sites, the genomic region 5 kb upstream to 1 kb downstream of the translation start site for each gene was searched using the FIMO package^17^ with default parameters. RNAseq data for decidualization is taken from Suahil, Y. et al. ^7^.

### Stromal invasion assay

Human dermal fibroblasts (HDFs, PCS-201-012, ATCC) were seeded at 60% confluency. NR2F6 gene was silenced using siRNA (IDT) with RNAiMAX (Thermo Fisher) following the manufacturer’s protocol for two days. A375 cells are labeled with Vybrant DiI (Thermo Fisher) and seeded in 96-well plate with nanopatterns at 40,000 cells/well. A cell free area in each well was created using BioTek AutoScratch (Agilent). HDFs were then detached and seeded into the scratched 96-wells (three wells per condition). Time-lapse images of A375 invading HDFs were taken using Zeiss Oberserver Z1 microscope. A375 invasion profile was analyzed using Fiji/ImageJ with wound healing size tool^18^. 2999 separate widths from each well were analyzed at time 0, and 24 h.

## Data Availability

All raw data in the figures will be made available on request. Sequencing data are available in NCBI GEO: (GSE197810 ID:200197810), and BioProject (PRJNA564062).

## References

[1] Wagner, G. P., Kshitiz, Dighe, A. & Levchenko, A. The Coevolution of Placentation and Cancer. *Annu Rev. Anim. Biosci.* **10**, 259–279 (2022). 34780249 10.1146/annurev-animal-020420-031544

[2] Kshitiz et al. Evolution of placental invasion and cancer metastasis are causally linked. *Nat. Ecol. Evol.* **3**, 1743–1753 (2019). 31768023 10.1038/s41559-019-1046-4 PMC7340496

[3] Suhail, Y. et al. Tracing the cis-regulatory changes underlying the endometrial control of placental invasion. *Proc. Natl Acad. Sci. USA* **119** (2022). 10.1073/pnas.2111256119 PMC8832988 35110402

[4] Dighe, A. et al. Experimental and phylogenetic evidence for correlated gene expression evolution in endometrial and skin fibroblasts. *iScience* **27**, 108593 (2024). 38174318 10.1016/j.isci.2023.108593 PMC10762354

[5] Suhail, Y., Afzal, J. & Kshitiz. Evolved Resistance to Placental Invasion Secondarily Confers Increased Survival in Melanoma Patients. *J. Clin. Med.* **10** (2021). 10.3390/jcm10040595 PMC7915120 33562461

[6] Wu, L., Stadtmauer, D. J., Maziarz, J. D. & Wagner, G. Decidual cell differentiation is evolutionarily derived from fibroblast activation. *bioRxiv* (2020).

[7] Suhail, Y. et al. Extravillous trophoblasts reverse the decidualization induced increase in matrix production by secreting TGFbeta antagonists Emilin-1 and Gremlin-1. *Cells Dev*, 203994 (2025). 10.1016/j.cdev.2025.203994 PMC11972693 39756583

[8] Afzal, J. et al. Paracrine HB-EGF signaling reduce enhanced contractile and energetic state of activated decidual fibroblasts by rebalancing SRF-MRTF-TCF transcriptional axis. *Front Cell Dev. Biol.* **10**, 927631 (2022). 36147738 10.3389/fcell.2022.927631 PMC9485834

[9] Suhail, Y., Maziarz, J., Dighe, A., Wagner, G. & Kshitiz. Cis-Regulatory Differences Explaining Evolved Levels of Endometrial Invasibility in Eutherian Mammals. *BioRxiv* (2020).

[10] Christian, M., Mak, I., White, J. O. & Brosens, J. J. Mechanisms of decidualization. *Reprod. Biomed. Online* **4**, 24–30 (2002). 12470561 10.1016/s1472-6483(12)60112-6

[11] Rytkonen, K. T. et al. Decidualization of Human Endometrial Stromal Fibroblasts is a Multiphasic Process Involving Distinct Transcriptional Programs. *Reprod. Sci.* **26**, 323–336 (2019). 30309298 10.1177/1933719118802056 PMC6728557

[12] Zorn, T. M., Bevilacqua, E. M. & Abrahamsohn, P. A. Collagen remodeling during decidualization in the mouse. *Cell Tissue Res* **244**, 443–448 (1986). 3719669 10.1007/BF00219220

[13] Peng, J. et al. Single-cell RNA-seq highlights intra-tumoral heterogeneity and malignant progression in pancreatic ductal adenocarcinoma. *Cell Res* **29**, 725–738 (2019). 31273297 10.1038/s41422-019-0195-y PMC6796938

[14] Zhang, X., Fang, B. & Huang, Y. F. Transcription factor binding sites are frequently under accelerated evolution in primates. *Nat. Commun.* **14**, 783 (2023). 36774380 10.1038/s41467-023-36421-3 PMC9922303

[15] Korotkevich, G. et al. Fast gene set enrichment analysis. *bioRxiv* (2021).

[16] Cancer Genome Atlas Research, N., Weinstein, J. N. et al. The Cancer Genome Atlas Pan-Cancer analysis project. *Nat. Genet* **45**, 1113–1120 (2013). 24071849 10.1038/ng.2764 PMC3919969

[17] Grant, C. E., Bailey, T. L. & Noble, W. S. FIMO: scanning for occurrences of a given motif. *Bioinformatics* **27**, 1017–1018 (2011). 21330290 10.1093/bioinformatics/btr064 PMC3065696

[18] Suarez-Arnedo, A. et al. An image J plugin for the high throughput image analysis of in vitro scratch wound healing assays. *PLoS One* **15**, e0232565 (2020). 32722676 10.1371/journal.pone.0232565 PMC7386569

